# Emerging multiscale insights on microbial carbon use efficiency in the land carbon cycle

**DOI:** 10.1038/s41467-024-52160-5

**Published:** 2024-09-13

**Authors:** Xianjin He, Elsa Abs, Steven D. Allison, Feng Tao, Yuanyuan Huang, Stefano Manzoni, Rose Abramoff, Elisa Bruni, Simon P. K. Bowring, Arjun Chakrawal, Philippe Ciais, Lars Elsgaard, Pierre Friedlingstein, Katerina Georgiou, Gustaf Hugelius, Lasse Busk Holm, Wei Li, Yiqi Luo, Gaëlle Marmasse, Naoise Nunan, Chunjing Qiu, Stephen Sitch, Ying-Ping Wang, Daniel S. Goll

**Affiliations:** 1 Laboratoire des Sciences du Climat et de l’Environnement, IPSL-LSCE, CEA/CNRS/UVSQ, Orme des Merisiers, Gif sur Yvette, France; 2 Department of Ecology and Evolutionary Biology, University of California Irvine, Irvine, CA USA; 3 Department of Earth System Science, University of California Irvine, Irvine, CA USA; 4 Department of Ecology and Evolutionary Biology, Cornell University, Ithaca, NY USA; 5 Key Laboratory of Ecosystem Network Observation and Modeling, Institute of Geographic Sciences and Natural Resources Research, Chinese Academy of Sciences, Beijing, China; 6 Department of Physical Geography and Bolin Centre for Climate Research, Stockholm University, Stockholm, Sweden; 7 Wintergreen Earth Science, Kennebunk, ME USA; 8 LG-ENS (Laboratoire de géologie) CNRS UMR 8538—Ecole normale supérieure, PSL University -IPSL, Paris, France; 9 Environmental Molecular Sciences Laboratory, Pacific Northwest National Laboratory, Richland, WA USA; 10 Department of Agroecology, Aarhus University, AU Viborg, Tjele Denmark; 11 iCLIMATE Interdisciplinary Centre for Climate Change, Aarhus University, Roskilde, Denmark; 12 Faculty of Environment, Science and Economy, University of Exeter, Exeter, UK; 13 Laboratoire de Météorologie Dynamique, Institut Pierre-Simon Laplace, CNRS, École Normale Supérieure, Université PSL, Sorbonne Université, École Polytechnique, Paris, France; 14 Physical and Life Sciences Directorate, Lawrence Livermore National Laboratory, Livermore, CA USA; 15 Department of Earth System Science, Ministry of Education Key Laboratory for Earth System Modeling, Institute for Global Change Studies, Tsinghua University, Beijing, China; 16 Soil and Crop Sciences Section, School of Integrative Plant Science, Cornell University, Ithaca, NY USA; 17 Ecole Normale Supérieure de Lyon, Lyon, France; 18 Institute of Ecology and Environmental Sciences—Paris, Sorbonne Université, CNRS, IRD, INRA, P7, UPEC, Paris, France; 19 Department of Soil and Environment, Swedish University of Agricultural Sciences, Uppsala, Sweden; 20 Research Center for Global Change and Complex Ecosystems, East China Normal University, Shanghai, China; 21 CSIRO Environment, Private Bag 10, Commonwealth Scientific and Industrial Research Organization, Clayton South, VIC 3169 Australia

**Keywords:** Carbon cycle, Carbon cycle, Carbon cycle, Ecological modelling

## Abstract

Microbial carbon use efficiency (CUE) affects the fate and storage of carbon in terrestrial ecosystems, but its global importance remains uncertain. Accurately modeling and predicting CUE on a global scale is challenging due to inconsistencies in measurement techniques and the complex interactions of climatic, edaphic, and biological factors across scales. The link between microbial CUE and soil organic carbon relies on the stabilization of microbial necromass within soil aggregates or its association with minerals, necessitating an integration of microbial and stabilization processes in modeling approaches. In this perspective, we propose a comprehensive framework that integrates diverse data sources, ranging from genomic information to traditional soil carbon assessments, to refine carbon cycle models by incorporating variations in CUE, thereby enhancing our understanding of the microbial contribution to carbon cycling.

## Abstract (web summary)

Microbial carbon use efficiency (CUE) is crucial for carbon storage, but its variability is difficult to capture due to inconsistent measurements and complex interactions. This perspective proposes integrating diverse data and models to improve CUE in carbon cycle models

## Introduction

Earth System Models (ESMs) are indispensable tools for predicting the planetary response to climate change^1^. The accuracy and reliability of ESMs are crucial for informing climate projections that guide policy decisions. Soils store more carbon (C) than plants, the surface ocean or the atmosphere, and thus are critical for the functioning of the Earth system^2^. While ESMs are becoming increasingly complex, their predictions of soil organic C (SOC) stocks have improved only marginally in recent decades^3,4^.

Microbial communities process most of the C entering the soil, thereby shaping its fate^5,6^. Microbes metabolize multiple C sources, including detritus, root exudates, and microbial metabolites^7^. The energy needed to acquire C depends on whether the compounds can be taken up directly or require prior enzymatic degradation^8^. Additionally, microbial community composition and functioning are influenced by prevailing climatic conditions^9–11^. The general omission of microbial community structure and related processes in C cycle models has been suggested as one of the causes for their poor performance in predicting SOC stocks and their responses to climate change^12,13^.

Recognizing the impracticality of representing every conceivable microbial metabolic pathway, many models combine a spectrum of microbial processes into a single metric referred to as microbial C use efficiency (CUE)^14,15^. CUE, as a model parameter or as a system property emerging from multiple co-occurring processes, represents the fraction of C uptake allocated to the production of new microbial biomass^16^. Using this definition, CUE declines as more C is used for respiration to generate energy (for substrate uptake, cellular maintenance, enzyme production) or for exudation (extracellular enzymes, polysaccharides)^17,18^. This pragmatic approach streamlines the modeling of soil C cycling by incorporating the diverse fates of microbial C, including biomass production, respiration, and exudation, thereby providing a more comprehensive understanding of microbially-mediated C-pathways.

**Fig. 1:**
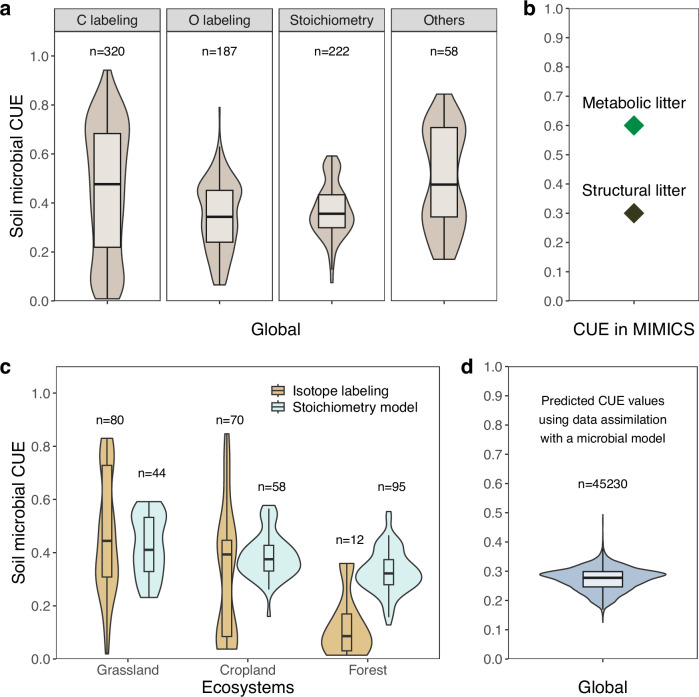
Variability of carbon use efficiency (CUE) at a global scale. **a** Observation-based CUE estimates at the global scale from C (^13^C and ^14^C) and ^18^O isotopic labeling, stoichiometric modeling and other methods. Data were collected from^19,21,49,95,114^. **b** CUE constants used in the MIcrobial-MIneral Carbon Stabilization model (MIMICS) for two litter types (diamonds). Metabolic litter comprises plant litter that decomposes easily, whereas structural litter is more resistant to decomposition^131^. **c** Observation-based estimates for different ecosystems using isotopic labeling^114^ or stoichiometric modeling^19^. **d** CUE values predicted using a microbial model assimilating information on SOC profiles^21^. Data assimilation integrates observed data into predictive models to refine model parameters and improve estimation accuracy.

However, accurately integrating the spatial or temporal dynamics of microbial CUE into soil C models remains a significant challenge. Most of the current C cycle models either lack explicit representation of CUE or treat it as a constant value^4^, despite our understanding that CUE varies under different environmental conditions. For example, observations indicate significant variability in CUE at the global scale^8^, which may be partially attributed to inconsistencies among measurement techniques (Fig. 1a). Moreover, comparisons across ecosystems reveal that CUE is generally higher in grasslands than in croplands, with forests consistently showing the lowest CUE values, regardless of the measurement approaches used^19,20^ (Fig. 1c). CUEs derived from data assimilation^21^ are also lower than those from more direct measurement approaches (Fig. 1d).

Several attempts have been made to reflect or incorporate CUE variations into models of litter^22^ or soil organic matter^9,13^ decomposition with the aim of assessing the implications for soil C cycling. For example, incorporating an empirically-derived negative relationship between microbial CUE and temperature into a microbial-explicit SOC model improved the simulation of contemporary soil C stocks^23^. Zhang et al.^24^ introduced the effects of substrate quality and soil fertility on microbial respiration, highlighting the joint control of litter quality and quantity on the steady-state SOC stocks. Wieder et al.^25^ enhanced the understanding of CUE variation by including two types of decomposers with differing substrate preferences and CUE (Fig. 1b). These examples suggest that more realistic representations of microbial C transformations have the scope for improving model predictions of soil C^23,26^. However, these predictions were poorly constrained by observational data, calling their reliability into question^21,27,28^.

In this Perspective, we synthesize our understanding of CUE regulatory factors and databases for constraining numerical models, with the aim of clarifying complexities, addressing controversies, and providing a holistic perspective on pathways to adequately reflect CUE variations in C cycle models and their consequences for simulated soil C stocks.

## Data availability and challenges

### Terminology and definitions of microbial CUE

**Fig. 2:**
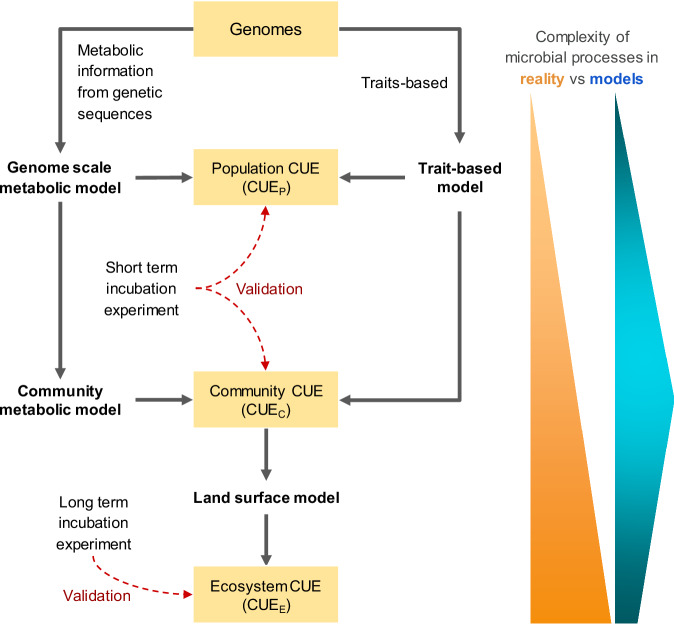
Schematic representation of a cluster of models integrating observational constraints on CUE at population (CUE_P_), community (CUE_C_) and ecosystem (CUE_E_) scales. The genome-scale metabolic model predicts the movement of metabolites within a cell based on its genomic information. CUE_P_ and CUE_C_ can be validated by short-term incubation measurements, while CUE_E_ requires long-term incubation measurements. Although the scales and processes governing CUE expand from individual cells to entire ecosystems, there is a practical limit to the extent they can be resolved in C cycle models.

The concept of microbial CUE, the fraction of C uptake that is used to produce microbial biomass^16–18^, is intuitively straightforward, but CUE definitions vary depending on the ecological processes involved, measurement methods, and scales of biological organization (e.g., population, community and ecosystem)^14,17^. Therefore, CUE can be regarded as an emergent parameter, encapsulating multiple processes within a single metric. It is useful in modeling as the number of processes that can be modeled is constrained by practical limitations (e.g., availability of data for calibration). Consequently, ecosystem models often simplify microbial process complexity, which in reality, escalates from the genomic to the ecosystem level (Fig. 2).

CUE is quantitatively expressed as the ratio of microbial growth (μ) to C uptake (U)^16,29^, that is, CUE = μ/U. This ratio encapsulates the efficiency with which microorganisms convert assimilated C into biomass. Microbial uptake involves C assimilation for growth (μ), respiration (R), and the secretion of extracellular enzymes and metabolites (EX). Geyer et al.^14^ introduced a nested conceptual framework for understanding CUE across different biological organization levels: population (CUE_P_), community (CUE_C_), and ecosystem (CUE_E_). This framework is useful for integrating C fluxes mediated by soil microbes into models at various ecological scales (Fig. 2).

CUE_P_ reflects the species-specific functioning of microbial taxa (e.g., biosynthesis rate, exudate production) and thermodynamics of C substrate metabolism that limits the proportion of C uptake used for biosynthesis versus C lost from the cell (e.g., mineralized or exuded as metabolites). Typically measured in cultured populations, the CUE_P_ formula adjusts for respiration (R) and exudation (EX) losses from the uptake, expressed as CUE_*P*_ = $\frac{U-R-{EX}}{U}$. CUE_C_ incorporates additional environmental and community factors influencing microbial metabolism in natural communities consisting of multiple populations. It focuses on gross microbial production prior to the recursive substrate recycling of necromass and exudates, capturing the metabolic response of microbial communities to substrates over short durations (hours), and is similarly expressed as CUE_*C*_ = $\frac{U-R-{EX}}{U}$.

CUE_E_ considers C retention as net microbial growth over longer time scales (days to months), taking into account the drivers of CUE_P_ and CUE_C_ as well as microbial biomass turnover. On these time scales, a significant proportion of microbial biomass is converted to necromass following microbial death (MD)^30^ such that CUE_*E*_ = $\frac{U-R-{EX}-{MD}}{U}$, encompassing all aspects of microbial C processing, including death and recycling processes.

### Methods for measuring microbial CUE

**Fig. 3:**
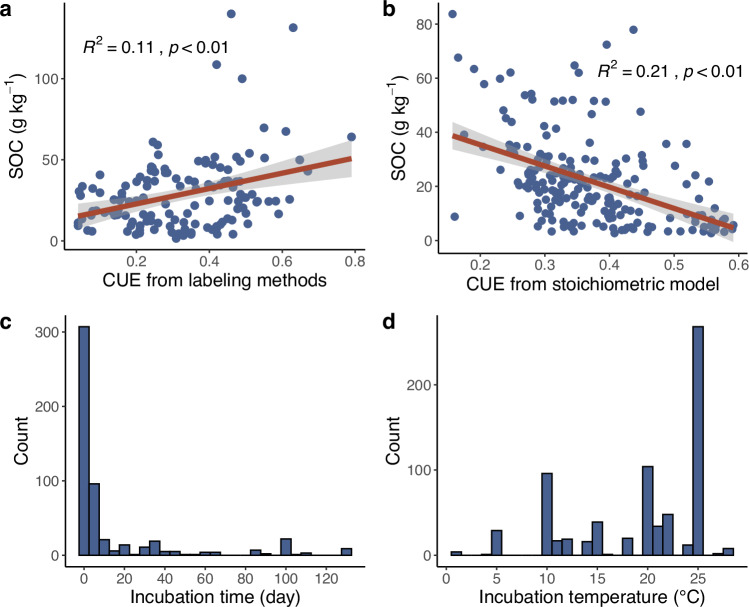
Impact of different research methods on the SOC-CUE relationship and variability in incubation conditions across studies. Panels **a** and **b** illustrate the relationships between soil organic carbon (SOC) concentration and CUE based on **a** isotopic labeling methods (^14^C, ^13^C-labeled substrates, and ^18^O water) and **b** stoichiometric modeling. Panel **c** presents the incubation durations, while panel **d** shows the temperatures employed in studies using labeling and incubation methods. Data sources: **a**^21^, **b**^19^, and **c**, **d**^20^.

Multiple approaches can be used to quantify CUE, such as isotopically labeling substrates^31,32^, stoichiometric modeling^22,33^ and others^34^. These methods rely on different assumptions and capture distinct microbial processes, which can explain the variability in CUE estimates across methods^8,35,36^ (Fig. 1a), including differences in the response of CUE to environmental changes^37^, and the relationship between CUE and SOC (Fig. 3a, b).

The most common approach for measuring CUE is the tracking of isotopically labeled compounds (^14^C, ^13^C labeled substrate, or ^18^O water) introduced to the system. Carbon isotopes in microbial substrates enable the differentiation between C allocated to microbial biomass and that released through respiration. Although this labeling technique is widely used, its results can be influenced by the choice and combination of substrates^31^, as well as the incubation period^14,38^. A significant limitation of this approach is that measured CUE reflects only the efficiency of those microbes that use the introduced substrates, not the entire microbial community. Furthermore, the variation in incubation times and temperatures across different studies (Fig. 3c, d) presents a substantial obstacle to standardizing CUE measurements.

The method using ^18^O-labeled water is based on the incorporation of the ^18^O-atom into microbial DNA as a measure of growth as compared to catabolic C losses as CO_2_^32,39^. This method has higher accuracy than the C labeling method as it is not substrate specific, does not perturb microbial metabolism like methods involving substrate addition, and exhibits comparatively less variability over time^35^. Nonetheless, this method faces limitations such as higher cost and demanding technical procedures. Concerns also arise regarding the method’s foundational assumptions, e.g., the presumption that water is the sole oxygen source for microbial DNA synthesis and the hypothesis that all microbial cells maintain a consistent DNA to biomass C ratio^40^. Furthermore, its applicability in dry soils is challenging^41^.

Stoichiometric modeling is a common method for indirectly estimating CUE, which is based on the assumption that microbes growing on plant detritus allocate C to produce enzymes and other necessary components to acquire nutrients in the appropriate elemental ratios at the whole-community scale^29,33^. This approach offers the advantage of requiring only a limited number of parameters, such as the activities of enzymes targeting C versus nitrogen (N) or phosphorus (P) acquisition and the C:N:P composition of the substrate and microbial biomass, which can be constrained by existing observations. However, it relies on highly simplified assumptions regarding elemental ratios and C allocation^36^. This approach inherently suggests lower CUE in soils with high SOC due to its focus on the metabolic costs of nutrient acquisition under conditions where nutrients are scarce relative to C. This outcome (Fig. 3b) starkly contrasts with the positive correlation between CUE and SOC observed using isotopic labeling techniques (Fig. 3a), which are commonly considered to provide a more realistic insight into the relationship between CUE and SOC. The isotope labeling method estimates microbial growth and CUE by tracking the incorporation of labeled atoms into biomass or DNA, reflecting intracellular biochemical transformations. In contrast, the stoichiometry model method estimates CUE by analyzing the activities of extracellular enzymes and the stoichiometric balance between organic matter and microbial biomass, focusing on extracellular metabolic processes^42^. Therefore, caution is advised when comparing results obtained from these two methods, even though they use the same term (CUE). We do not yet know the extent to which the stoichiometric and isotope methods are comparable. Until we understand which patterns can be accurately captured by the simpler stoichiometric method, we should rely on the more robust ^18^O method for measuring actual CUE and the ^13^C method for CUE associated with specific substrates.

In addition to the methods mentioned above, there are other less commonly used approaches, including the use of ^18^O in water vapor to minimize impact on soil moisture^41^, metabolic flux analysis^17^, and calorespirometry^43^. Each method offers unique advantages and faces specific limitations, grounded in their underlying assumptions and theoretical bases^35–37^. These limitations not only affect the accuracy of these methods but also introduce significant comparability issues. Consequently, there is an urgent need to improve current methodologies and integrate innovative techniques to more accurately assess soil microbial CUE.

### Data gap

Given the methodological challenges in measuring CUE in situ, field assessments of microbial CUE are rare. The vast majority of existing CUE observations have been obtained from lab incubations. Yet, these CUE observations remain scarce at the global scale, a situation which is exacerbated by the lack of harmonization of observations from different measurement approaches. For some ecosystems, observations are few or even nonexistent, including ecosystems that play a critical role in the global C cycle, such as tropical rainforests, wetlands, and peatlands^44,45^.

Existing CUE measurements mostly come from studies of the litter and surface mineral soil^16^. Thus, our understanding of microbial CUE in subsurface soil remains limited, which is problematic as large amounts of C are stored in subsoils globally, and especially those of wetlands and peatlands. The few existing studies indicate that microbial CUE decreases with soil depth^46,47^ and that subsurface CUE may be less sensitive to warming^31^ but more sensitive to nutrient variations^48^.

Moreover, data on temporal variations in CUE are lacking. A commonly overlooked factor that may contribute significantly to CUE variability in soil ecosystems, regardless of methodology, is seasonality in CUE. Seasonal changes are associated with significant variations in substrate availability, temperature and moisture, all of which may have a substantial impact on the growth and respiration of soil microorganisms, thereby altering microbial CUE^39^. For example, CUE estimated using the ^18^O incorporation method ranged from 0.1 to 0.7 in soils from an agricultural field site and from 0.1 to 0.6 at a forest site within one year^49^. It has also been reported that soil microbial CUE exhibits significant fluctuations within a short period (daily) after rewetting^50,51^. This temporal dynamic in CUE values could contribute to the significant variability observed in CUE measurements.

## Regulatory factors governing microbial CUE

**Fig. 4:**
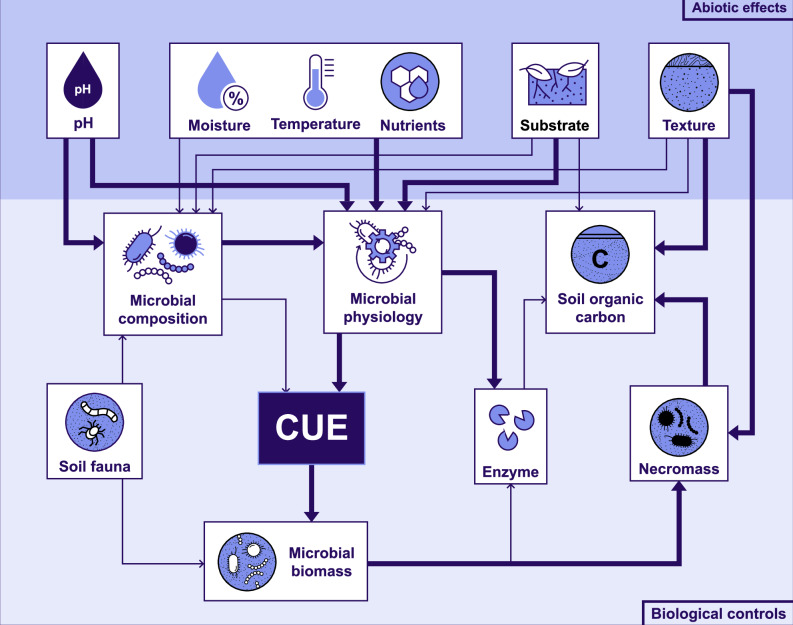
Framework of biological and abiotic determinants of CUE in a carbon cycle context. The darker-colored area in the figure indicates biological controls; the lighter-colored area indicates abiotic effects. The arrows depict implicit relationships and the width of the arrows corresponds to the levels of scientific certainty: confident assertions are represented by thick lines, while less confident assertions are indicated by thinner lines. These confidence levels are based on the expertise of the authors.

The incorporation of soil microbial CUE dynamics into process-based models necessitates a comprehensive understanding of a range of regulatory factors influencing CUE (Fig. 4). CUE at a specific biological level is influenced by features of both the microbial community itself (biological controls) and its external environment (abiotic controls). These factors frequently interact, particularly at the community and ecosystem levels: abiotic controls can modify CUE_C_ or CUE_E_ by regulating biological controls, while biological controls may induce adaptation to abiotic factors, thereby influencing the impact of abiotic controls.

### Biological controls

#### Microbial physiological state

Microbial CUE reflects the physiological state of microorganisms. Under natural conditions, only a small proportion (values vary from 1% to >20% in different studies^52,53^) of soil microbial cells are metabolically active, and soil respiration primarily originates from these metabolically active cells^53^. Nonetheless, a high fraction of microbial cells in the soil are in a potentially active state (10 to 60% of the total microbial biomass), meaning that they are ready to start using available substrates within a few hours after easily available substrate is added. The shifts in physiological states of these microbial cells, resulting from changes in temperature, moisture, or substrate availability, significantly impact CUE^54^. Consequently, CUE_P_ or CUE_C_ measurement methods relying on substrate addition may overestimate CUE^14^, and shifts in physiological state can lead to seasonal variations in CUE^49^.

### Microbial community diversity and composition

Increased microbial diversity enriches the spectrum of metabolic functions within a community, potentially leading to greater microbial growth^55^ and CUE_C_ by facilitating more efficient use of varied C sources^10,56^. The composition of microbial communities, notably the ratio of fungal to bacterial biomass (F:B), plays a critical role in determining CUE_C_^57^. Communities dominated by fungi can show higher CUE_C_, attributed to their higher biomass C to N) ratios (C:N) and their proficiency in decomposing complex organic materials^58^, or lower CUE due to the high costs associated with resource acquisition by decomposer fungi^57^. Therefore, this contrasting evidence from plant litter studies indicates that the relationship between F:B ratio and CUE is context-dependent^57,59^. Alternatively, an approach categorizing microorganisms into copiotrophs (*r*-strategists with low CUE) versus oligotrophs (*K*-strategists with high CUE) has been promising for estimating CUE^60^. For example, shifts from *r*-strategists to *K*-strategists explain increased CUE_C_ along a successional gradient in the southeastern Tibetan Plateau^61^.

Changes in community composition may also enable microbial communities to alter their CUE in response to environmental changes or fluctuations^62,63^. For instance, long-term warming experiments indicate a decline in the temperature sensitivity of CUE_C_, suggesting that shifts in microbial composition can maintain CUE_C_ despite changes in temperature and substrate quality^31^. Similarly, modeling studies suggest that changing microbial community composition can reduce the sensitivity of CUE_C_ to substrate quality^64^ and soil moisture fluctuations^65^.

### Biotic interactions

In the soil food web, biotic interactions such as mutualism, facilitation, competition, and predation can shape CUE_C_^56^. Interspecific microbial competition drives accelerated growth rates, accompanied by the release of secondary metabolites that can negatively affect CUE_C_^66^. Antagonistic interactions may trigger stress responses, further diminishing CUE_C_^67^. Conversely, facilitation enhances CUE_C_ by broadening species-realized niches, alleviating environmental stress, and reducing extracellular enzyme production costs^64^. Biotic interactions at higher trophic levels, such as predation, can variably affect CUE_C_ by altering microbial density and influencing the outcomes of interspecific competition^68,69^.

### Abiotic controls

#### Temperature

Temperature significantly affects soil microbial CUE, with respiration often increasing more than growth in short-term incubations, resulting in a decrease in CUE_P_^9,34,70^. The impact on CUE_C_ and CUE_E_ is less clear^63^, likely due to varied responses among microbial taxa^71,72^ and interactive effects with other environmental factors^38,39,46,73^. Temperature shifts can lead to changes in community traits or select for taxa with distinct life strategies, known as trait modification and trait filtering, respectively^74,75^. However, limited research on how CUE_P_ varies among different taxa in response to temperature impairs our ability to accurately predict changes in CUE_C_^76–78^.

The interplay between direct and indirect temperature effects on soil microbial CUE_C_ and CUE_E_ complicates our understanding of the impact of warming on CUE. Warming can intensify C-nutrient imbalances, potentially diminishing microbial CUE^79^, but it can also improve the efficiency of substrate utilization, thereby enhancing CUE^32,72^. Expected reductions in soil moisture due to increased evapotranspiration under warming conditions^80^ add another layer of complexity, with the combined impacts of temperature and moisture on microbial CUE remaining inadequately explored^10,81^. Some soil C models, including Millennial^82^ and MIMICS^25^ have begun to account for the temperature dependency of CUE_C_, indicating a growing recognition of the importance of including the dynamic response of microbial CUE to fluctuations in temperature.

### Soil water availability

Increased soil moisture promotes microbial growth and CUE by improving substrate diffusivity and accessibility, and lowering investment in osmolyte synthesis, as long as conditions remain oxic^8,10,83^. Prolonged water stress reduces soil substrate accessibility and increases the need to synthesize osmolytes to survive during dry periods, leading to lower CUE_C_^83^, even though the taxa that remain active in dry conditions can maintain relatively high growth rates^84^. Furthermore, drought reduces plant C inputs to the soil^83^, thus potentially leaving microbes with fewer lower resources, resulting in lower CUE. The intricate interplay of drought-induced changes in microbial respiration and growth may leave CUE unchanged if the affected processes balance each other^78^. High levels of soil moisture may also reduce microbial CUE. As soil pores fill with water, air spaces and oxygen diffusivity decline, potentially leading to anaerobic conditions if saturation occurs. Under O_2_ limitation, soil microbes shift from aerobic to anaerobic respiration or fermentation, significantly reducing energy yield and leading to decreased microbial growth and CUE while having little impact on CO_2_ production rate due to upregulated biochemical rates^83^.

Microbial responses to rewetting of a dry soil also cause rapid changes in CUE, as shown in modeling studies^50^ and confirmed by empirical evidence^51^. Upon rewetting, respiration increases while growth lags behind, especially when the soil has been dry for a long period^51^. As a result, just after rewetting, CUE is low and then increases as growth recovers during the first days after rewetting. However, after this initial pulse of microbial activity, CUE peaks and decreases again as substrates released during rewetting are consumed^51^.

### Nutrient availability

The availability of nutrients such as N and P significantly affects microbial growth and respiration according to the concept of stoichiometric homeostasis which assumes constrained biomass C:N:P ratios of microbial cells^29,64^. Consequently, CUE decreases with increasing substrate C-to-nutrient ratios and increases with nutrient amendment when organic substrates are nutrient-poor^22,29^. Several C cycle models, such as the one proposed by Manzoni et al.^85^ and its later implementation^24^, have integrated CUE dynamics as a function of stoichiometry. In contrast to the homeostasis concept, recent findings highlight the capability of microbes to store and use nutrients dynamically, contributing to a stable CUE across different environments by separating growth and respiration processes from immediate nutrient availability^86^. This resilience to nutrient stress suggests that future C modeling should incorporate microbial nutrient storage dynamics for enhanced predictive accuracy.

### Soil pH

Soil pH influences microbial CUE_C_ and CUE_E_ by affecting the bacterial community composition and acting as a potential stressor^87^. It also impacts CUE by altering microbial community composition^88^, nutrient solubility^83^, and metal toxicity (e.g., aluminum^87^). Habitats with neutral pH generally have higher bacterial diversity and biomass compared to acidic or alkaline soils^7^. The response of community composition to a shift in soil pH from acidic to neutral corresponded with a significant increase in CUE_C_^87,89^. However, recent research indicates a complex interplay between soil pH, microbial community composition, and CUE dynamics, evidenced by both negative correlations^90^ and a U-shaped response curve, pinpointing a critical threshold at pH 6.4^91^, although the calculations to document this are complex and may necessitate refinement.

### Soil texture and structure

Microbial growth is intricately linked to substrate accessibility, which is influenced by soil environmental conditions like texture and soil structure. Approximately 40–70% of soil bacteria are associated with microaggregates and clay particles^92^. The structural complexity of the soil environment also plays a crucial role in shaping the community structure and function of soil microorganisms at the ecosystem level^93^. Heterogeneity of soil structure and composition creates diverse microhabitats that influence microbial interactions, diversity, distributions, and activity, as well as ecosystem processes like nutrient cycling and organic matter decomposition^94^. Still, limited information exists on the relationship between soil texture or structure and microbial CUE. A recent meta-analysis found a significant positive link between microbial CUE_C_ or CUE_E_ for glucose and soil clay content^95^, which was attributed to increased clay content enhancing substrate adsorption^96^, thereby limiting substrate availability to microbes^97^, and resulting in higher microbial CUE_C_ or CUE_E_.

### Substrate quality

Substrate quality, defined by the chemical characteristics of organic matter that influence its decomposability, such as the C:N ratio and molecular composition, significantly impacts soil microbial CUE^98^. A “high-quality” substrate typically has a lower C:N ratio, indicating a balanced N content relative to C, and a lower content of recalcitrant compounds, which generally leads to faster decomposition and higher CUE by providing C and nutrients that microbes require for growth and metabolism^8^. Compounds requiring multiple enzymatic steps for degradation can lead to reduced efficiency in building biomass. Polymeric substrates like lignin and cellulose need depolymerization before cellular uptake, whereas smaller substrates readily diffuse across membranes^62^. Takriti et al. (2018) found a positive association between soil CUE_C_ and ratios of cellulase to phenol oxidase enzyme activity potential, which was considered to be indicative of soil organic matter (SOM) substrate quality^46^. Different substrates necessitate distinct metabolic pathways, resulting in different respiration rates per unit C assimilated^8,99^. Frey et al. (2013) observed lower microbial CUE_C_ when soils were amended with oxalic acid or phenolic compounds compared to glucose, despite similar molecular sizes^31^.

Microbial CUE increases with the chemical energy per mole of C in the substrate, highlighting the importance of substrate chemistry for microbial CUE variability in soil^8^. This relationship is akin to the concept of energetic imbalance^100^, which parallels the idea of stoichiometric imbalance. The energy content of soil microbial biomass and substrate can be quantified by the degree of reduction (γ), which refers to the average number of electrons available per C atom for biochemical reactions, indicating the energy density of the substrate or biomass^8^. The degree of reduction of soil microbial biomass (γ_B_) is typically around 4.2, while that of substrate (γ_S_) usually varies between 1 (e.g., for oxalate) and 8 (methane)^8^. Most of the substrates used by soil microorganisms have a γ_S_ of 3 (e.g., various organic acids), 4 (e.g., glucose and other carbohydrates), and rarely 5 or higher (e.g., leucine, polyhydroxyalkanoates or lipids)^8^. When γ_S_ is lower than γ_B_, the substrate’s energy content is insufficient to meet microbial demand, necessitating the oxidation of more substrate per unit of C assimilated, thereby reducing CUE^101^. These insights form the basis of the stoichiometric modeling for indirect CUE estimates.

## SOC-CUE relationship

The relationship between CUE and SOC concentration at the ecosystem level can be positive, negative, or non-existent, depending on the interactions among multiple processes^21,92,96,102–104^. Higher CUE can lead to increased SOC through biosynthesis and accumulation of microbial by-products—facilitating SOC formation via the entombing effect^16,102,105^ — or conversely, trigger SOC decline through the priming effect by ramping up microbial biomass and enzyme activity^9^. While some studies suggest a negative correlation between CUE and SOC^103,104,106^, the majority of research supports a positive relationship^21,74,107,108^, indicating that higher CUE is often linked to increased SOC levels. In a recent study, Tao et al.^21^ employed observational data and data assimilation algorithms and found that, on a global scale, CUE is positively correlated with SOC concentration, arguing for CUE as the major determinant for SOC formation. However, subsequent arguments have raised methodological concerns which might have obscured the importance of microbial community dynamics^27^ and SOC stabilization processes^109^.

Indeed, the link between microbial CUE and SOC is contingent upon the stabilization of microbial necromass within soil aggregates or its association with minerals^96,102,105^. This stabilization process, pivotal for enhancing SOC, is significantly influenced by physico-chemical soil properties, which vary greatly and determine the potential for necromass protection^110,111^. Positive SOC-CUE relationships could be anticipated in soils with high physicochemical C stabilization potential and microbial communities that convert simple chemical substrates into necromass^111^. Conversely, when soil microbes face environmental stress, the relationship between CUE and SOC becomes less predictable. Particularly under conditions where nutrients are limited relative to carbon, the increased microbial respiration required to maintain stoichiometric balance leads to a decreased CUE^29,33^. Further reductions in CUE may be driven by environmental challenges such as low oxygen or pH^88,106^, as well as the physiological costs of microbial competition^66^. However, these stressors on microbial activity may differently affect SOC, potentially leading to either a negative or negligible correlation between CUE and SOC^106^. It’s worth noting that in organic-rich soils, such as peat, C stabilization relies more on the accumulation of undecomposed plant material than on necromass formation^112^, making the link between CUE and SOC less direct. Therefore, the CUE-SOC relationship in organic soils is expected to differ from mineral soils where C is mainly stabilized by mineral associations.

**Fig. 5:**
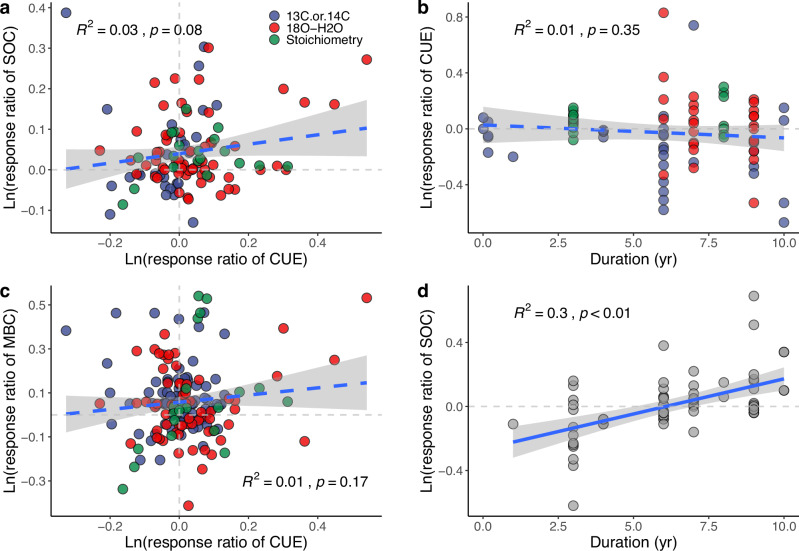
Contrasting responses of SOC and CUE to fertilization. Correlations between ln-transformed response ratios of microbial CUE and ln-transformed response ratios of (**a**) SOC and (**c**) microbial biomass C (MBC); and the correlation between experiment duration and ln-transformed response ratios of (**b**) CUE and (**d**) SOC. The response ratio is calculated as the ratio of the measured value in treatment to the value in the control. Data are from meta-analyses^27,37,114^. Both datasets include observations from all three methods of CUE measurement, i.e., C labeling, O labeling, and stoichiometry modeling as indicated by symbol colors in **a**–**c**.

Additionally, it is important to recognize the distinct sensitivities of microbial CUE and SOC to environmental changes, as their responses are not synchronized. Microbial CUE can adjust rapidly, from days to months, in contrast to SOC, which may take years or even decades to respond to a measurable extent^49,113^. Data from two meta-analyses highlight this disparity, showing that although fertilization positively affects both CUE_C_ and SOC^37,114^, the response ratios of CUE_C_ were not significantly correlated with the response ratios of SOC, or even microbial biomass C content (Fig. 5a, c). Here, the “response ratio” is calculated as the ratio of the measured value in the treatment to the value in the control. Furthermore, the response ratios of microbial CUE_C_ were not significantly related to treatment duration (within ten years of treatment) (Fig. 5b), whereas the response ratios of SOC increased significantly with experiment duration (Fig. 5d). Therefore, SOC gradually approaches a new equilibrium over several decades, whereas CUE achieves equilibrium almost immediately. This discrepancy underscores the importance of considering the state (SOC and microbial biomass) dynamics of an ecosystem when evaluating the interplay between microbial CUE and SOC dynamics.

### Using models and data across scales to clarify the microbial role in C cycling

#### Integrating genomic data with CUE and C models

With the rise of high throughput sequencing technology, the use of genomic datasets to help calibrate or validate C models has become both feasible and affordable. This capacity is especially valuable when predicting CUE^115^. As genomic data related to microbial traits becomes more readily available at both the population^116^ and community levels through metagenomics^117^, there is a growing need to effectively integrate this data into C cycle models. This integration requires models that can handle complex microbial interactions, from individual populations to entire communities (Fig. 2).

One way to integrate genomic data is by converting the genetic sequences of microbes into information on metabolic pathways (e.g., cellulose degradation, lignin degradation, nitrogen reduction, and fermentation) using genome-scale metabolic models (GEMs)^118^. GEMs take into account the microbe’s environment, such as substrate availability, and predict the transformation of metabolites within a cell based on its genomic information. This process allows for the calculation of CUE at the population level by analyzing substrate use and CO_2_ production^118^. For community-level CUE, GEMs can be combined into microbial community models that simulate interactions between different microbial taxa: The ‘computation of microbial ecosystems in time and space metabolic modeling platform’ (COMETS) extends GEMs to include dynamics of microbial growth and interactions, providing a tool for predicting CUE_C_ under various environmental conditions^115^.

An alternative modeling approach at the community level is based on traits (e.g., quantity of cellulase produced, maximum rate of reaction (V_max_) of cellulose decay by cellulase, V_max_ of cellulose-monomer uptake, and turnover rate), such as the DEMENT model, which uses data on microbial traits to simulate substrate use and CO_2_ production^119^. This model can predict both CUE_P_ and CUE_C_ under different environmental conditions and over time. However, translating genomic data into traits remains challenging^120^. Genomic datasets typically indicate the presence or absence of certain genes or pathways, but additional information, such as that from GEMs or experimental data, is necessary to accurately map these genes to functional traits in the models.

Validating genomic and trait-based models is crucial and can be achieved using community-level genomic datasets, which offer insights into microbial strategies that affect CUE, such as nutrient recycling and stress tolerance^117,121^. Combining these models with traditional CUE measurements and omics data allows for the creation of detailed maps of community-level CUE, offering new insights into C cycling dynamics and providing input information for C cycle models.

A major challenge in this field is the high computational demand of integrating omic data into complex models. One solution is the development of computational emulators that can simulate the dynamics of microbial models more efficiently, bridging the gap between detailed, small-scale models and broader applications in C cycle studies^122^. This approach promises to improve our understanding of microbial contributions to C cycling, leveraging the power of genomic data to inform and validate complex ESMs.

### Harmonization of CUE measurements and aligning measured and modeled CUE

Harmonizing soil microbial CUE measurements across different methods, i.e., aligning results from different methodologies, poses a challenge due to the differences across measurement techniques. While adopting a universal protocol for CUE measurement—a single, standardized measurement method— would be ideal, it may not be feasible given the complexities of CUE. Therefore, a more practical approach involves providing a clear and comprehensive description of the methodologies used in different studies. This detailed reporting should include information on the physiological processes considered, such as maintenance, enzyme production, biomass generation, and mortality rates. This level of detail helps in understanding and comparing results across studies, as well as in selecting appropriate data for model calibration^17^.

In contemporary soil C models that explicitly incorporate microbial processes^25,82^, the CUE is close to empirically measured CUE_*C*_. To achieve a uniform approach to CUE measurement, microbial models that resolve key processes influencing CUE, such as uptake, respiration, exudation, and microbial death could be used^17^. Such models can generate CUE metrics that align with different measurement methodologies by incorporating a complete or partial set of these processes into their calculations. Furthermore, these models can be adapted to conduct numerical experiments with specific substrates or to incorporate isotopic tracers (e.g., ^13^C, ^14^C, ^18^O) to simulate outcomes from labeling experiments. This adaptability allows for the exploration of hypotheses regarding discrepancies in measurements under diverse conditions by modifying model boundary conditions. Additionally, microbial models serve as foundational tools for integrating microbial metabolism into broader global C models, potentially enhanced by machine learning emulators for improved scalability and applicability.

### Constraining CUE using model-data fusion

Data assimilation encompasses a collection of techniques, including Bayesian inference, that refine biogeochemical models by integrating observational data. This process not only updates model parameters to reflect the most likely values based on available data but also quantifies their uncertainties, thus bridging the gap between empirical observations and theoretical models^107^. This approach is particularly valuable for parameters like microbial CUE, which are challenging to measure directly in the field due to technical limitations. An innovative application of data assimilation is demonstrated by Tao et al.^21^, who developed the PROcess-guided deep learning and DAta-driven (PRODA) approach^123,124^. This method integrates global-scale SOC data with a microbially explicit model to produce a global map of microbial CUE. PRODA employs traditional Bayesian data assimilation to estimate parameters at specific sites and then uses deep learning to extrapolate these site-specific parameter estimates to a global scale. The result is a set of parameters that optimally align with observed data, offering a detailed view of microbial CUE and SOC storage patterns worldwide, along with other soil C cycle dynamics such as decomposition rates, environmental impacts on soil respiration, and vertical C transport^21^.

Despite the potential of approaches like PRODA to harness large datasets for enhancing our understanding of the soil C cycle, their computational intensity—stemming from the extensive data sampling required by Bayesian inference—may limit their application in models with complex structures. The next wave of data assimilation techniques will likely integrate process-based models with deep learning algorithms more seamlessly^121^. Such advancements could offer quicker parameter optimization and facilitate comparisons across different models, paving the way for more accurate and comprehensive assessments of microbial CUE and C cycle dynamics on a global scale.

### Long-term SOC records and ecosystem manipulation experiments

Ecosystem manipulation experiments and observations of natural gradients offer invaluable insights into how microbial communities and CUE adapt to global change factors. Especially insightful are field experiments (or studies leveraging natural gradients) that alter environmental factors such as soil temperature, precipitation patterns, or nutrient levels^76,125^ over long durations. These experiments provide critical data on the enduring effects of global change drivers on CUE, while simultaneously highlighting the limitations of current models and enhancing our comprehension of ecological processes. Integrating the results from these experiments with model simulations, supported by proven site modeling protocols and extra observational data, is crucial for steadily enhancing the accuracy and complexity of models^126^.

Incorporating radiocarbon (^14^C) data and long-term SOC records into models is also vital for refining CUE forecasts across longer (decadal to centennial) time scales. This temporal information is essential for capturing the dynamics of CUE over time, thereby improving the precision of models in depicting spatial and temporal fluctuations^127^.

### Diagnosing CUE from existing models or simulation archives

In global C modeling, approaches to quantify the environmental impact on organic matter decomposition and stabilization differ significantly. An effective method for estimating microbial CUE at the ecosystem level as emerging from model simulations involves the calculation of the ratio between soil heterotrophic respiration (R) and gross decomposition (D) within these models. Gross decomposition refers to the sum of all C fluxes transferred between the modeled soil C pools that are mediated by microbial processes, excluding physically mediated transfers (e.g., sorption, aggregation, or leaching). This includes all C removed from organic matter pools, whether it is lost as CO_2_ or transferred to another pool (SI-Text 1). This ratio effectively quantifies microbial-mediated C losses from SOC pools, integrating both growth (anabolic processes) and respiration (catabolic processes). Under steady-state conditions, it is assumed that heterotrophic respiration aligns with microbial C uptake, resulting in the formula: CUE = 1 - R/D. The steady-state assumption implies that microbial communities and SOC stock are stable in time (i.e., in equilibrium with boundary conditions). This is an approximation of real systems where SOC varies due to anthropogenic and natural changes (e.g., Holocene climatic variations). This diagnosed CUE, emerging as a property inherent to the model, is not susceptible to the equifinality issues that can affect the underlying intrinsic model parameters (like CUE_C_), and it does not necessitate the incorporation of explicitly microbial models, offering a simplified yet insightful metric. These model-based CUE estimates, derived from long-term flux averages (e.g., 20 years), represent stable C stocks. In contrast, measurement-based estimates, taken over shorter periods, are more susceptible to significant CUE variations due to asynchronous fluctuations in components such as respiration and degradation, potentially introducing estimation inaccuracies. This timescale discrepancy likely accounts for the greater variability observed in measurement-based CUE compared to model-based CUE. We propose this “model-diagnosed CUE” as a novel metric, designed to estimate microbial CUE from model outputs without direct measurements of microbial uptake.

Analyzing diagnosed CUE and its relationship with SOC across various models, such as those evaluated in the Trends in the land carbon cycle (TRENDY) model intercomparison project^2^, facilitates the identification of differences attributable to unique model structures and assumptions. For example, warming-induced CO_2_ emissions should be higher in models with low diagnosed CUE compared to high CUE as the warming-induced stimulation of microbial activity will result in relatively more C being respired than cycled within the soil systems. This approach further allows the benchmarking and subsequent refinement of diagnosed CUE estimates using observed CUE_E_ data.

**Fig. 6:**
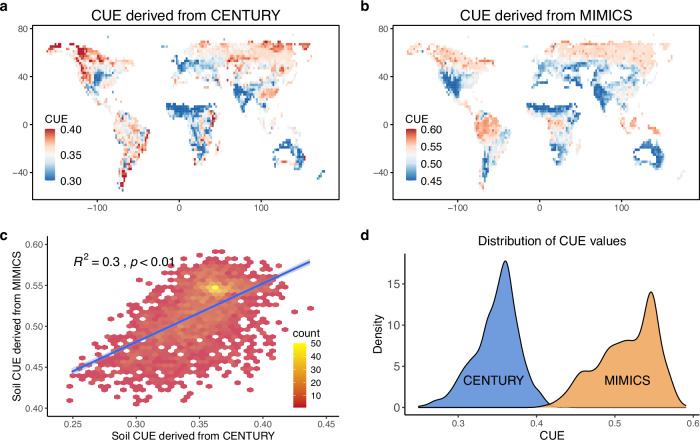
Diagnosed CUE from two existing soil C models. CUE diagnosed from a nutrient-enabled version of the the Organizing Carbon and Hydrology In Dynamic Ecosystems land surface model (ORCHIDEE-CNP) deploying a soil module based on (**a**) the CENTURY model^128^, or (**b**) the MIMICS model with constant intrinsic CUE_C_^132^. **c** Correlation between diagnosed CUE values from the CENTURY-based model and the MIMICS-based model. **d** Distribution frequency of CUE for the two scenarios.

For instance, we derived CUE estimates from simulations conducted with two different versions of the Organizing Carbon and Hydrology In Dynamic Ecosystems (ORCHIDEE) land surface model^128^, which differ in the SOC model deployed. The CENTURY SOC model (Fig. S1), which is widely used but does not resolve microbial processes, uses first-order decay, while the MIMICS model (Fig. S2) resolves microbial physiology, providing a more mechanistic understanding of microbial processes. The resulting global CUE maps (the average of simulation results over 20 consecutive years) revealed significant spatial variability (Fig. 6a, b). While the two maps showed a good correlation (Fig. 6c), the CUE values diagnosed from the MIMICS model were higher than those from the CENTURY model (Fig. 6d). These findings underscore the importance of incorporating observational data into model calibration efforts to enhance the accuracy and reliability of SOC predictions by realistically resolving CUE.

In conclusion, the inherent structure of a model significantly shapes its outcomes, making the integration of empirical data with data-constrained models a fundamental step toward realistic predictions^129,130^. Precisely delineating the spatial and temporal dynamics of CUE in models that specifically address microbial activities is crucial for the reliability of their predictions of SOC status and dynamics. Moreover, future soil C models must navigate the intricate balance between the complex regulatory mechanisms of CUE, other processes governing SOC formation and stabilization, and the practicality of model use to promote more precise projections of CUE responses under diverse environmental scenarios. This Perspective underscores the importance of combining different data sources with sophisticated modeling techniques to refine global CUE predictions. By incorporating genomic data, standardizing measurement protocols, applying data assimilation practices and critically evaluating CUE within existing frameworks, our comprehension of the global dynamics of microbial CUE can be markedly improved. This Perspective provides a roadmap for establishing an effective modeling approach to accurately represent global soil microbial CUE and its interactions with other biological and abiotic processes that regulate SOC dynamics.

## Supplementary information

Supplementary information

## References

[1] Eyring, V. et al. Overview of the Coupled Model Intercomparison Project Phase 6 (CMIP6) experimental design and organization. *Geosci. Model Dev.* **9**, 1937–1958 (2016). 10.5194/gmd-9-1937-2016

[2] Friedlingstein, P. et al. Global carbon budget 2023. *Earth Syst. Sci. Data* **15**, 5301–5369 (2023). 10.5194/essd-15-5301-2023

[3] Shi, Z. et al. Global‐scale convergence obscures inconsistencies in soil carbon change predicted by earth system models. *AGU Adv.* **5**, e2023AV001068 (2024). 10.1029/2023AV001068

[4] Varney, R. M., Chadburn, S. E., Burke, E. J. & Cox, P. M. Evaluation of soil carbon simulation in CMIP6 Earth system models. *Biogeosciences* **19**, 4671–4704 (2022). 10.5194/bg-19-4671-2022

[5] Crowther, T. W. et al. The global soil community and its influence on biogeochemistry. *Science* **365**, eaav0550 (2019). 31439761 10.1126/science.aav0550

[6] Ranheim Sveen, T., Hannula, S. E. & Bahram, M. Microbial regulation of feedbacks to ecosystem change. *Trends Microbiol*. S0966842X23001919 (2023) 10.1016/j.tim.2023.06.006 (2023). 10.1016/j.tim.2023.06.006 37500365

[7] Fierer, N. Embracing the unknown: disentangling the complexities of the soil microbiome. *Nat. Rev. Microbiol.* **15**, 579–590 (2017). 28824177 10.1038/nrmicro.2017.87

[8] Manzoni, S., Taylor, P., Richter, A., Porporato, A. & Ågren, G. I. Environmental and stoichiometric controls on microbial carbon‐use efficiency in soils. *New Phytol.* **196**, 79–91 (2012). 22924405 10.1111/j.1469-8137.2012.04225.x

[9] Allison, S. D., Wallenstein, M. D. & Bradford, M. A. Soil-carbon response to warming dependent on microbial physiology. *Nat. Geosci.* **3**, 336–340 (2010). 10.1038/ngeo846

[10] Domeignoz-Horta, L. A. et al. Microbial diversity drives carbon use efficiency in a model soil. *Nat. Commun.* **11**, 3684 (2020). 32703952 10.1038/s41467-020-17502-z PMC7378083

[11] Karhu, K. et al. Temperature sensitivity of soil respiration rates enhanced by microbial community response. *Nature* **513**, 81–84 (2014). 25186902 10.1038/nature13604

[12] Luo, Z. et al. Convergent modelling of past soil organic carbon stocks but divergent projections. *Biogeosciences* **12**, 4373–4383 (2015). 10.5194/bg-12-4373-2015

[13] Wieder, W. R., Cleveland, C. C., Smith, W. K. & Todd-Brown, K. Future productivity and carbon storage limited by terrestrial nutrient availability. *Nat. Geosci.* **8**, 441–444 (2015). 10.1038/ngeo2413

[14] Geyer, K. M., Kyker-Snowman, E., Grandy, A. S. & Frey, S. D. Microbial carbon use efficiency: accounting for population, community, and ecosystem-scale controls over the fate of metabolized organic matter. *Biogeochemistry* **127**, 173–188 (2016). 10.1007/s10533-016-0191-y

[15] Treseder, K. K. et al. Integrating microbial ecology into ecosystem models: challenges and priorities. *Biogeochemistry* **109**, 7–18 (2012). 10.1007/s10533-011-9636-5

[16] Manzoni, S. et al. Reviews and syntheses: carbon use efficiency from organisms to ecosystems—definitions, theories, and empirical evidence. *Biogeosciences* **15**, 5929–5949 (2018). 10.5194/bg-15-5929-2018

[17] Dijkstra, P. et al. On maintenance and metabolisms in soil microbial communities. *Plant Soil* **476**, 385–396 (2022). 10.1007/s11104-022-05382-9

[18] Hagerty, S. B., Allison, S. D. & Schimel, J. P. Evaluating soil microbial carbon use efficiency explicitly as a function of cellular processes: implications for measurements and models. *Biogeochemistry* **140**, 269–283 (2018). 10.1007/s10533-018-0489-z

[19] He, P., Zhang, Y., Shen, Q., Ling, N. & Nan, Z. Microbial carbon use efficiency in different ecosystems: a meta-analysis based on a biogeochemical equilibrium model. *Glob. Change Biol.* **00**, 1–17 (2023). 10.1111/gcb.16861 37431700

[20] Qiao, Y. et al. Global variation of soil microbial carbon-use efficiency in relation to growth temperature and substrate supply. *Sci. Rep.* **9**, 5621 (2019). 30948759 10.1038/s41598-019-42145-6 PMC6449510

[21] Tao, F. et al. Microbial carbon use efficiency promotes global soil carbon storage. *Nature*10.1038/s41586-023-06042-3 (2023). 10.1038/s41586-023-06042-3 PMC10307633 37225998

[22] Manzoni, S. Flexible carbon-use efficiency across litter types and during decomposition partly compensates nutrient imbalances—results from analytical stoichiometric models. *Front. Microbiol.* **8**, 661 (2017). 28491054 10.3389/fmicb.2017.00661 PMC5405148

[23] Wieder, W. R., Bonan, G. B. & Allison, S. D. Global soil carbon projections are improved by modelling microbial processes. *Nat. Clim. Change* **3**, 909–912 (2013). 10.1038/nclimate1951

[24] Zhang, H. et al. Modeling the effects of litter stoichiometry and soil mineral N availability on soil organic matter formation using CENTURY-CUE (v1.0). *Geosci. Model Dev.* **11**, 4779–4796 (2018). 10.5194/gmd-11-4779-2018

[25] Wieder, W. R., Grandy, A. S., Kallenbach, C. M. & Bonan, G. B. Integrating microbial physiology and physio-chemical principles in soils with the MIcrobial-MIneral Carbon Stabilization (MIMICS) model. *Biogeosciences* **11**, 3899–3917 (2014). 10.5194/bg-11-3899-2014

[26] Sulman, B. N., Phillips, R. P., Oishi, A. C., Shevliakova, E. & Pacala, S. W. Microbe-driven turnover offsets mineral-mediated storage of soil carbon under elevated CO2. *Nat. Clim. Change* **4**, 1099–1102 (2014). 10.1038/nclimate2436

[27] He, X. et al. Model uncertainty obscures major driver of soil carbon. *Nature* **627**, E1–E3 (2024). 38448702 10.1038/s41586-023-06999-1

[28] Shi, Z., Crowell, S., Luo, Y. & Moore, B. Model structures amplify uncertainty in predicted soil carbon responses to climate change. *Nat. Commun.* **9**, 2171 (2018). 29867087 10.1038/s41467-018-04526-9 PMC5986763

[29] Sinsabaugh, R. L. et al. Stoichiometry of microbial carbon use efficiency in soils. *Ecol. Monogr.* **86**, 172–189 (2016). 10.1890/15-2110.1

[30] Camenzind, T., Mason-Jones, K., Mansour, I., Rillig, M. C. & Lehmann, J. Formation of necromass-derived soil organic carbon determined by microbial death pathways. *Nat. Geosci.* **16**, 115–122 (2023). 10.1038/s41561-022-01100-3

[31] Frey, S. D., Lee, J., Melillo, J. M. & Six, J. The temperature response of soil microbial efficiency and its feedback to climate. *Nat. Clim. Change* **3**, 395–398 (2013). 10.1038/nclimate1796

[32] Spohn, M., Klaus, K., Wanek, W. & Richter, A. Microbial carbon use efficiency and biomass turnover times depending on soil depth—implications for carbon cycling. *Soil Biol. Biochem.* **96**, 74–81 (2016). 10.1016/j.soilbio.2016.01.016

[33] Sinsabaugh, R. L., Manzoni, S., Moorhead, D. L. & Richter, A. Carbon use efficiency of microbial communities: stoichiometry, methodology and modelling. *Ecol. Lett.* **16**, 930–939 (2013). 23627730 10.1111/ele.12113

[34] Zhang, Q., Qin, W., Feng, J. & Zhu, B. Responses of soil microbial carbon use efficiency to warming: review and prospects. *Soil Ecol. Lett.* **4**, 307–318 (2022). 10.1007/s42832-022-0137-3

[35] Geyer, K. M., Dijkstra, P., Sinsabaugh, R. & Frey, S. D. Clarifying the interpretation of carbon use efficiency in soil through methods comparison. *Soil Biol. Biochem.* **128**, 79–88 (2019). 10.1016/j.soilbio.2018.09.036

[36] Schimel, J., Weintraub, M. N. & Moorhead, D. Estimating microbial carbon use efficiency in soil: Isotope-based and enzyme-based methods measure fundamentally different aspects of microbial resource use. *Soil Biol. Biochem.* **169**, 108677 (2022). 10.1016/j.soilbio.2022.108677

[37] Hu, J., Huang, C., Zhou, S. & Kuzyakov, Y. Nitrogen addition to soil affects microbial carbon use efficiency: meta‐analysis of similarities and differences in ^13^ C and ^18^ O approaches. *Glob. Change Biol.* **28**, 4977–4988 (2022). 10.1111/gcb.16226 35617026

[38] Hagerty, S. B. et al. Accelerated microbial turnover but constant growth efficiency with warming in soil. *Nat. Clim. Change* **4**, 903–906 (2014). 10.1038/nclimate2361

[39] Simon, E. et al. Microbial growth and carbon use efficiency show seasonal responses in a multifactorial climate change experiment. *Commun. Biol.* **3**, 584 (2020). 33067550 10.1038/s42003-020-01317-1 PMC7567817

[40] Qu, L., Wang, C. & Bai, E. Evaluation of the 18O-H2O incubation method for measurement of soil microbial carbon use efficiency. *Soil Biol. Biochem.* **145**, 107802 (2020). 10.1016/j.soilbio.2020.107802

[41] Canarini, A. et al. Quantifying microbial growth and carbon use efficiency in dry soil environments via ^18^ O water vapor equilibration. *Glob. Change Biol.* **26**, 5333–5341 (2020). 10.1111/gcb.15168 PMC7497233 32472728

[42] Sun, L. et al. Interpreting the differences in microbial carbon and nitrogen use efficiencies estimated by 18O labeling and ecoenzyme stoichiometry. *Geoderma* **444**, 116856 (2024). 10.1016/j.geoderma.2024.116856

[43] Yang, S. et al. Enhancing insights: exploring the information content of calorespirometric ratio in dynamic soil microbial growth processes through calorimetry. *Front. Microbiol.* **15**, 1321059 (2024). 38371938 10.3389/fmicb.2024.1321059 PMC10869564

[44] Fewster, R. E. et al. Imminent loss of climate space for permafrost peatlands in Europe and Western Siberia. *Nat. Clim. Change* **12**, 373–379 (2022). 10.1038/s41558-022-01296-7

[45] Hugelius, G. et al. Large stocks of peatland carbon and nitrogen are vulnerable to permafrost thaw. *Proc. Natl. Acad. Sci. USA* **117**, 20438–20446 (2020). 32778585 10.1073/pnas.1916387117 PMC7456150

[46] Takriti, M. et al. Soil organic matter quality exerts a stronger control than stoichiometry on microbial substrate use efficiency along a latitudinal transect. *Soil Biol. Biochem.* **121**, 212–220 (2018). 10.1016/j.soilbio.2018.02.022

[47] Zhang, Q. et al. Whole-soil-profile warming does not change microbial carbon use efficiency in surface and deep soils. *Proc. Natl. Acad. Sci. USA* **120**, e2302190120 (2023). 37523548 10.1073/pnas.2302190120 PMC10410710

[48] Jiang, Y. et al. Deep soil microbial carbon use efficiency responds stronger to nitrogen deposition than top soil in tropical forests, southern China. *Plant Soil*10.1007/s11104-024-06509-w (2024).

[49] Schnecker, J. et al. Seasonal dynamics of soil microbial growth, respiration, biomass, and carbon use efficiency in temperate soils. *Geoderma* **440**, 116693 (2023). 10.1016/j.geoderma.2023.116693

[50] Brangarí, A. C., Manzoni, S. & Rousk, J. A soil microbial model to analyze decoupled microbial growth and respiration during soil drying and rewetting. *Soil Biol. Biochem.* **148**, 107871 (2020). 10.1016/j.soilbio.2020.107871

[51] Li, X., Leizeaga, A., Rousk, J., Hugelius, G. & Manzoni, S. Drying intensity and acidity slow down microbial growth recovery after rewetting dry soils. *Soil Biol. Biochem.* **184**, 109115 (2023). 10.1016/j.soilbio.2023.109115

[52] Couradeau, E. et al. Probing the active fraction of soil microbiomes using BONCAT-FACS. *Nat. Commun.* **10**, 2770 (2019). 31235780 10.1038/s41467-019-10542-0 PMC6591230

[53] Blagodatskaya, E. & Kuzyakov, Y. Active microorganisms in soil: critical review of estimation criteria and approaches. *Soil Biol. Biochem.* **67**, 192–211 (2013). 10.1016/j.soilbio.2013.08.024

[54] Hasby, F. A., Barbi, F., Manzoni, S. & Lindahl, B. D. Transcriptomic markers of fungal growth, respiration and carbon-use efficiency. *FEMS Microbiol. Lett.* **368**, fnab100 (2021). 34338746 10.1093/femsle/fnab100 PMC8374604

[55] Khurana, S. et al. Interactive effects of microbial functional diversity and carbon availability on decomposition—a theoretical exploration. *Ecol. Model.* **486**, 110507 (2023). 10.1016/j.ecolmodel.2023.110507

[56] Anthony, M. A., Crowther, T. W., Maynard, D. S., Van Den Hoogen, J. & Averill, C. Distinct assembly processes and microbial communities constrain soil organic carbon formation. *One Earth* **2**, 349–360 (2020). 10.1016/j.oneear.2020.03.006

[57] Soares, M. & Rousk, J. Microbial growth and carbon use efficiency in soil: Links to fungal-bacterial dominance, SOC-quality and stoichiometry. *Soil Biol. Biochem.* **131**, 195–205 (2019). 10.1016/j.soilbio.2019.01.010

[58] Malik, A. A. et al. Soil fungal:bacterial ratios are linked to altered carbon cycling. *Front. Microbiol*. **7**, 1247 (2016). 10.3389/fmicb.2016.01247 PMC4977315 27555839

[59] Keiblinger, K. M. et al. The effect of resource quantity and resource stoichiometry on microbial carbon-use-efficiency: resource quantity/quality drives microbial C-use-efficiency. *FEMS Microbiol. Ecol*. 10.1111/j.1574-6941.2010.00912.x (2010). 10.1111/j.1574-6941.2010.00912.x 20550579

[60] Six, J., Frey, S. D., Thiet, R. K., & Batten, K. M. Bacterial and fungal contributions to carbon sequestration in agroecosystems. *Soil Sci. Soc. Am. J.* **70**, 555–569 (2006). 10.2136/sssaj2004.0347

[61] Ma, S., Zhu, W., Wang, W., Li, X. & Sheng, Z. Microbial assemblies with distinct trophic strategies drive changes in soil microbial carbon use efficiency along vegetation primary succession in a glacier retreat area of the southeastern Tibetan Plateau. *Sci. Total Environ.* **867**, 161587 (2023). 36638988 10.1016/j.scitotenv.2023.161587

[62] Allison, S. D. Modeling adaptation of carbon use efficiency in microbial communities. *Front. Microbiol*. **5**, 571 (2014). 10.3389/fmicb.2014.00571 PMC4211550 25389423

[63] Qu, L. et al. Stronger compensatory thermal adaptation of soil microbial respiration with higher substrate availability. *ISME J*. wrae025. 10.1093/ismejo/wrae025 (2024). 10.1093/ismejo/wrae025 PMC10945366 38366058

[64] Kaiser, C., Franklin, O., Dieckmann, U. & Richter, A. Microbial community dynamics alleviate stoichiometric constraints during litter decay. *Ecol. Lett.* **17**, 680–690 (2014). 24628731 10.1111/ele.12269 PMC4315898

[65] Brangarí, A. C., Manzoni, S. & Rousk, J. The mechanisms underpinning microbial resilience to drying and rewetting—a model analysis. *Soil Biol. Biochem.* **162**, 108400 (2021). 10.1016/j.soilbio.2021.108400

[66] Maynard, D. S., Crowther, T. W. & Bradford, M. A. Fungal interactions reduce carbon use efficiency. *Ecol. Lett.* **20**, 1034–1042 (2017). 28677157 10.1111/ele.12801

[67] Iven, H., Walker, T. W. N. & Anthony, M. Biotic interactions in soil are underestimated drivers of microbial carbon use efficiency. *Curr. Microbiol.* **80**, 13 (2023). 10.1007/s00284-022-02979-2 PMC9718865 36459292

[68] Frey, S. D. Protozoan grazing affects estimates of carbon utilization efficiency of the soil microbial community. *Soil Biol. Biochem*. **33**, 1759–1768 (2001).

[69] Ma, L. et al. Long-term conservation tillage enhances microbial carbon use efficiency by altering multitrophic interactions in soil. *Sci. Total Environ.* **915**, 170018 (2024). 38224879 10.1016/j.scitotenv.2024.170018

[70] Tian, W. et al. Thermal adaptation occurs in the respiration and growth of widely distributed bacteria. *Glob. Change Biol.* **28**, 2820–2829 (2022). 10.1111/gcb.16102 35090074

[71] Pold, G. et al. Carbon use efficiency and its temperature sensitivity covary in soil bacteria. *mBio* **11**, e02293–19 (2020). 31964725 10.1128/mBio.02293-19 PMC6974560

[72] Tian, J. et al. Microbially mediated mechanisms underlie soil carbon accrual by conservation agriculture under decade-long warming. *Nat. Commun.* **15**, 377 (2024). 38191568 10.1038/s41467-023-44647-4 PMC10774409

[73] Walker, T. W. N. et al. Microbial temperature sensitivity and biomass change explain soil carbon loss with warming. *Nat. Clim. Change* **8**, 885–889 (2018). 10.1038/s41558-018-0259-x PMC6166784 30288176

[74] Kallenbach, C. M., Wallenstein, M. D., Schipanksi, M. E. & Grandy, A. S. Managing agroecosystems for soil microbial carbon use efficiency: ecological unknowns, potential outcomes, and a path forward. *Front. Microbiol.* **10**, 1146 (2019). 31178846 10.3389/fmicb.2019.01146 PMC6543778

[75] Ye, J., Bradford, M. A., Maestre, F. T., Li, F. & García‐Palacios, P. Compensatory thermal adaptation of soil microbial respiration rates in global croplands. *Glob. Biogeochem. Cycles* **34**, e2019GB006507 (2020).

[76] Metze, D. et al. Soil warming increases the number of growing bacterial taxa but not their growth rates. *Sci. Adv.* **10**, eadk6295 (2024). 38394199 10.1126/sciadv.adk6295 PMC10889357

[77] Saifuddin, M., Bhatnagar, J. M., Segrè, D. & Finzi, A. C. Microbial carbon use efficiency predicted from genome-scale metabolic models. *Nat. Commun.* **10**, 3568 (2019). 31395870 10.1038/s41467-019-11488-z PMC6687798

[78] Smith, T. P., Clegg, T., Bell, T. & Pawar, S. Systematic variation in the temperature dependence of bacterial carbon use efficiency. *Ecol. Lett.* **24**, 2123–2133 (2021). 34240797 10.1111/ele.13840

[79] Sun, Y. et al. A global meta-analysis on the responses of C and N concentrations to warming in terrestrial ecosystems. *CATENA* **208**, 105762 (2022). 10.1016/j.catena.2021.105762

[80] Xu, W. et al. A meta-analysis of the response of soil moisture to experimental warming. *Environ. Res. Lett.* **8**, 044027 (2013). 10.1088/1748-9326/8/4/044027

[81] Fuchslueger, L. et al. Microbial carbon and nitrogen cycling responses to drought and temperature in differently managed mountain grasslands. *Soil Biol. Biochem.* **135**, 144–153 (2019). 10.1016/j.soilbio.2019.05.002

[82] Abramoff, R. Z. et al. Improved global-scale predictions of soil carbon stocks with Millennial Version 2. *Soil Biol. Biochem.* **164**, 108466 (2022). 10.1016/j.soilbio.2021.108466

[83] Zheng, Q. et al. Growth explains microbial carbon use efficiency across soils differing in land use and geology. *Soil Biol. Biochem.* **128**, 45–55 (2019). 31579288 10.1016/j.soilbio.2018.10.006 PMC6774786

[84] Metze, D. et al. Microbial growth under drought is confined to distinct taxa and modified by potential future climate conditions. *Nat. Commun.* **14**, 5895 (2023). 37736743 10.1038/s41467-023-41524-y PMC10516970

[85] Manzoni, S. Optimal metabolic regulation along resource stoichiometry gradients. *Ecol. Lett*. **20**, 1182–1191 (2017). 10.1111/ele.12815 28756629

[86] Mason-Jones, K., Breidenbach, A., Dyckmans, J., Banfield, C. C. & Dippold, M. A. Intracellular carbon storage by microorganisms is an overlooked pathway of biomass growth. *Nat. Commun.* **14**, 2240 (2023). 37076457 10.1038/s41467-023-37713-4 PMC10115882

[87] Jones, D. L., Cooledge, E. C., Hoyle, F. C., Griffiths, R. I. & Murphy, D. V. pH and exchangeable aluminum are major regulators of microbial energy flow and carbon use efficiency in soil microbial communities. *Soil Biol. Biochem.* **138**, 107584 (2019). 10.1016/j.soilbio.2019.107584

[88] Malik, A. A. et al. Land use driven change in soil pH affects microbial carbon cycling processes. *Nat. Commun.* **9**, 3591 (2018). 30181597 10.1038/s41467-018-05980-1 PMC6123395

[89] Silva-Sánchez, A., Soares, M. & Rousk, J. Testing the dependence of microbial growth and carbon use efficiency on nitrogen availability, pH, and organic matter quality. *Soil Biol. Biochem.* **134**, 25–35 (2019). 10.1016/j.soilbio.2019.03.008

[90] Zhang, X. et al. Erosion effects on soil microbial carbon use efficiency in the mollisol cropland in northeast China. *Soil Ecol. Lett.* **5**, 230176 (2023). 10.1007/s42832-023-0176-4

[91] Schroeder, J. et al. Liming effects on microbial carbon use efficiency and its potential consequences for soil organic carbon stocks. *Soil Biol. Biochem.* **191**, 109342 (2024). 10.1016/j.soilbio.2024.109342

[92] Schmidt, M. W. I. et al. Persistence of soil organic matter as an ecosystem property. *Nature* **478**, 49–56 (2011). 21979045 10.1038/nature10386

[93] Young, I. M. & Crawford, J. W. Interactions and self-organization in the soil-microbe complex. *Science* **304**, 1634–1637 (2004). 15192219 10.1126/science.1097394

[94] Kuzyakov, Y. & Blagodatskaya, E. Microbial hotspots and hot moments in soil: concept & review. *Soil Biol. Biochem.* **83**, 184–199 (2015). 10.1016/j.soilbio.2015.01.025

[95] Islam, Md. R., Singh, B. & Dijkstra, F. A. Microbial carbon use efficiency of glucose varies with soil clay content: a meta-analysis. *Appl. Soil Ecol.* **181**, 104636 (2023). 10.1016/j.apsoil.2022.104636

[96] Cai, Y. et al. Assessing the accumulation efficiency of various microbial carbon components in soils of different minerals. *Geoderma* **407**, 115562 (2022). 10.1016/j.geoderma.2021.115562

[97] Jeewani, P. H. et al. The stoichiometric C-Fe ratio regulates glucose mineralization and stabilization via microbial processes. *Geoderma* **383**, 114769 (2021). 10.1016/j.geoderma.2020.114769

[98] Bölscher, T., Wadsö, L., Börjesson, G. & Herrmann, A. M. Differences in substrate use efficiency: impacts of microbial community composition, land use management, and substrate complexity. *Biol. Fertil. Soils* **52**, 547–559 (2016). 10.1007/s00374-016-1097-5

[99] Jones, D. L. et al. Role of substrate supply on microbial carbon use efficiency and its role in interpreting soil microbial community-level physiological profiles (CLPP). *Soil Biol. Biochem.* **123**, 1–6 (2018). 10.1016/j.soilbio.2018.04.014

[100] Chakrawal, A., Calabrese, S., Herrmann, A. M. & Manzoni, S. Interacting bioenergetic and stoichiometric controls on microbial growth. *Front. Microbiol.* **13**, 859063 (2022). 35656001 10.3389/fmicb.2022.859063 PMC9152356

[101] Kleerebezem, R. & Van Loosdrecht, M. C. M. A generalized method for thermodynamic state analysis of environmental systems. *Crit. Rev. Environ. Sci. Technol.* **40**, 1–54 (2010). 10.1080/10643380802000974

[102] Cotrufo, M. F., Wallenstein, M. D., Boot, C. M., Denef, K. & Paul, E. The Microbial Efficiency-Matrix Stabilization (MEMS) framework integrates plant litter decomposition with soil organic matter stabilization: do labile plant inputs form stable soil organic matter? *Glob. Change Biol.* **19**, 988–995 (2013). 10.1111/gcb.12113 23504877

[103] Craig, M. E. et al. Fast-decaying plant litter enhances soil carbon in temperate forests but not through microbial physiological traits. *Nat. Commun.* **13**, 1229 (2022). 35264580 10.1038/s41467-022-28715-9 PMC8907208

[104] Sokol, N. W. et al. The path from root input to mineral-associated soil carbon is shaped by habitat-specific microbial traits and soil moisture. *Soil Biol. Biochem*. 109367 10.1016/j.soilbio.2024.109367 (2024).

[105] Liang, C., Schimel, J. P. & Jastrow, J. D. The importance of anabolism in microbial control over soil carbon storage. *Nat. Microbiol.* **2**, 17105 (2017). 28741607 10.1038/nmicrobiol.2017.105

[106] Li, Z. et al. Microbial metabolic capacity regulates the accrual of mineral-associated organic carbon in subtropical paddy soils. *Soil Biol. Biochem*. 109457 10.1016/j.soilbio.2024.109457 (2024).

[107] Luo, Y. & Schuur, E. A. G. Model parameterization to represent processes at unresolved scales and changing properties of evolving systems. *Glob. Change Biol.* **26**, 1109–1117 (2020). 10.1111/gcb.14939 31782216

[108] Kallenbach, C. M., Frey, S. D. & Grandy, A. S. Direct evidence for microbial-derived soil organic matter formation and its ecophysiological controls. *Nat. Commun.* **7**, 13630 (2016). 27892466 10.1038/ncomms13630 PMC5133697

[109] Xiao, K.-Q. et al. Beyond microbial carbon use efficiency. *Natl. Sci. Rev*. nwae059 10.1093/nsr/nwae059 (2024). 10.1093/nsr/nwae059 PMC10939436 38487496

[110] Georgiou, K. et al. Global stocks and capacity of mineral-associated soil organic carbon. *Nat. Commun.* **13**, 3797 (2022). 35778395 10.1038/s41467-022-31540-9 PMC9249731

[111] Zhu, E. et al. Enhanced mineral preservation rather than microbial residue production dictates the accrual of mineral‐associated organic carbon along a weathering gradient. *Geophys. Res. Lett.* **51**, e2024GL108466 (2024). 10.1029/2024GL108466

[112] García-Palacios, P. et al. Dominance of particulate organic carbon in top mineral soils in cold regions. *Nat. Geosci*. 10.1038/s41561-023-01354-5 (2024).

[113] Lí, J. et al. Subarctic winter warming promotes soil microbial resilience to freeze–thaw cycles and enhances the microbial carbon use efficiency. *Glob. Change Biol.* **30**, e17040 (2024). 10.1111/gcb.17040 38273522

[114] Wu, J., Cheng, X. & Liu, G. Increased soil organic carbon response to fertilization is associated with increasing microbial carbon use efficiency: data synthesis. *Soil Biol. Biochem.* **171**, 108731 (2022). 10.1016/j.soilbio.2022.108731

[115] Dukovski, I. et al. A metabolic modeling platform for the computation of microbial ecosystems in time and space (COMETS). *Nat. Protoc.* **16**, 5030–5082 (2021). 34635859 10.1038/s41596-021-00593-3 PMC10824140

[116] Karaoz, U. & Brodie, E. L. microTrait: a toolset for a trait-based representation of microbial genomes. *Front. Bioinforma.* **2**, 918853 (2022). 10.3389/fbinf.2022.918853 PMC9580909 36304272

[117] Piton, G. et al. Life history strategies of soil bacterial communities across global terrestrial biomes. *Nat. Microbiol.* **8**, 2093–2102 (2023). 37798477 10.1038/s41564-023-01465-0

[118] Gu, C., Kim, G. B., Kim, W. J., Kim, H. U. & Lee, S. Y. Current status and applications of genome-scale metabolic models. *Genome Biol.* **20**, 121 (2019). 31196170 10.1186/s13059-019-1730-3 PMC6567666

[119] Abs, E., Albright, M. B. N. & Allison, S. D. Invasions eliminate the legacy effects of substrate history on microbial nitrogen cycling. *Ecosphere* **15**, e4754 (2024). 10.1002/ecs2.4754

[120] Bernstein, D. B., Sulheim, S., Almaas, E. & Segrè, D. Addressing uncertainty in genome-scale metabolic model reconstruction and analysis. *Genome Biol.* **22**, 64 (2021). 33602294 10.1186/s13059-021-02289-z PMC7890832

[121] Malik, A. A. et al. Defining trait-based microbial strategies with consequences for soil carbon cycling under climate change. *ISME J.* **14**, 1–9 (2020). 31554911 10.1038/s41396-019-0510-0 PMC6908601

[122] Demirer, E. et al. Improving the performance of reactive transport simulations using artificial neural networks. *Transp. Porous Media* **149**, 271–297 (2023). 10.1007/s11242-022-01856-7

[123] Tao, F. et al. Deep learning optimizes data-driven representation of soil organic carbon in earth system model over the conterminous United States. *Front. Big Data* **3**, 17 (2020). 33693391 10.3389/fdata.2020.00017 PMC7931903

[124] Reichstein, M. et al. Deep learning and process understanding for data-driven Earth system science. *Nature* **566**, 195–204 (2019). 30760912 10.1038/s41586-019-0912-1

[125] Song, J. et al. A meta-analysis of 1,119 manipulative experiments on terrestrial carbon-cycling responses to global change. *Nat. Ecol. Evol.* **3**, 1309–1320 (2019). 31427733 10.1038/s41559-019-0958-3

[126] Norby, R. J. et al. Model–data synthesis for the next generation of forest free‐air CO_2_FACE experiments. *New Phytol.* **209**, 17–28 (2016). 10.1111/nph.13593 26249015

[127] Tifafi, M. et al. The use of radiocarbon 14C to constrain carbon dynamics in the soil module of the land surface model ORCHIDEE (SVN r5165). *Geosci. Model Dev.* **11**, 4711–4726 (2018). 10.5194/gmd-11-4711-2018

[128] Goll, D. S. et al. A representation of the phosphorus cycle for ORCHIDEE (revision 4520). *Geosci. Model Dev.* **10**, 3745–3770 (2017). 10.5194/gmd-10-3745-2017

[129] Luo, Y. et al. Toward more realistic projections of soil carbon dynamics by Earth system models. *Glob. Biogeochem. Cycles* **30**, 40–56 (2016). 10.1002/2015GB005239

[130] Tao, F. et al. Convergence in simulating global soil organic carbon by structurally different models after data assimilation. *Glob. Change Biol.* **30**, e17297 (2024). 10.1111/gcb.17297 38738805

[131] Wieder, W. R., Grandy, A. S., Kallenbach, C. M., Taylor, P. G. & Bonan, G. B. Representing life in the Earth system with soil microbial functional traits in the MIMICS model. *Geosci. Model Dev.* **8**, 1789–1808 (2015). 10.5194/gmd-8-1789-2015

[132] Zhang, H. et al. Microbial dynamics and soil physicochemical properties explain large‐scale variations in soil organic carbon. *Glob. Change Biol.* **26**, 2668–2685 (2020). 10.1111/gcb.14994 31926046

