# Supplementary material for: Emerging multiscale insights on microbial carbon use efficiency in the land carbon cycle
Source: Nat Commun. 2024 Sep 13;15:8010. doi: 10.1038/s41467-024-52160-5 (PMC11399347; doi:10.1038/s41467-024-52160-5)
Supplement: Supplementary file 1 — Supplementary information [file 41467_2024_52160_MOESM1_ESM.pdf]

Supplementary information for Emerging multiscale insights on microbial carbon use efficiency in the land carbon cycle

Xianjin He[1], Elsa Abs[1], Steven D. Allison[2,3], Feng Tao[4], Yuanyuan Huang[5], Stefano Manzoni[6], Rose Abramoff[7], Elisa Bruni[8], Simon P.K. Bowring[1], Arjun Chakrawal[9], Philippe Ciais[1], Lars Elsgaard[10,11], Pierre Friedlingstein[12,13], Katerina Georgiou[14], Gustaf Hugelius[6], Lasse Busk Holm[10], Wei Li[15], Yiqi Luo[16], Gaëlle Marmasse[1,17], Naoise Nunan[18,19], Chunjing Qiu[20], Stephen Sitch[12], Ying-Ping Wang[21], Daniel S. Goll[1,*]

1 Laboratoire des Sciences du Climat et de l'Environnement, IPSL-LSCE, CEA/CNRS/UVSQ, Orme des Merisiers, 91191, Gif sur Yvette, France.

2 Department of Ecology and Evolutionary Biology, University of California Irvine, Irvine, CA, 92697, USA.

3 Department of Earth System Science, University of California Irvine, Irvine, CA, 92697, USA.

4 Department of Ecology and Evolutionary Biology, Cornell University, Ithaca, NY, 14850, USA.

5 Key Laboratory of Ecosystem Network Observation and Modeling, Institute of Geographic Sciences and Natural Resources Research, Chinese Academy of Sciences, Beijing, 100101, China.

6 Department of Physical Geography and Bolin Centre for Climate Research, Stockholm University, Stockholm SE-10691, Sweden.

7 Wintergreen Earth Science, Kennebunk, ME, USA.

8 LG-ENS (Laboratoire de géologie) CNRS UMR 8538 - Ecole normale supérieure, PSL University -IPSL, Paris, France.

9 Environmental Molecular Sciences Laboratory, Pacific Northwest National Laboratory, Richland, WA 99354, USA.

10 Department of Agroecology, Aarhus University, 8830 Tjele, Denmark.

11 iCLIMATE Interdisciplinary Centre for Climate Change, Aarhus University, 4000 Roskilde, Denmark.

12 Faculty of Environment, Science and Economy, University of Exeter, Exeter, EX4 4QF, UK.

13 Laboratoire de Météorologie Dynamique, Institut Pierre-Simon Laplace, CNRS, École Normale Supérieure, Université PSL, Sorbonne Université, École Polytechnique, Paris, France.

14 Physical and Life Sciences Directorate, Lawrence Livermore National Laboratory, Livermore, CA 94551, USA.

15 Department of Earth System Science, Ministry of Education Key Laboratory for Earth System Modeling, Institute for Global Change Studies, Tsinghua University, Beijing, China.

16 Soil and Crop Sciences Section, School of Integrative Plant Science, Cornell University, Ithaca, NY 14850, USA.

17 Ecole Normale Supérieure de Lyon, 69342 Lyon, France.

18 Institute of Ecology and Environmental Sciences – Paris, Sorbonne Université, CNRS, IRD, INRA, P7, UPEC, 4 place Jussieu, 75005 Paris, France.

19 Department of Soil and Environment, Swedish University of Agricultural Sciences, 75007 Uppsala, Sweden.

20 Research Center for Global Change and Complex Ecosystems, East China Normal University, Shanghai, China.

21 CSIRO Environment, Private Bag 10, Commonwealth Scientific and Industrial Research Organization, Clayton South, VIC 3168 Australia.

* Corresponding author: Daniel S. Goll (dsgoll123@gmail.com)

Text 1. The definition of model-diagnosed microbial carbon use efficiency (CUE):

We propose "model-diagnosed CUE" as a novel metric, designed to estimate microbial CUE from the output of SOC models without direct measurements of microbial uptake. In steady-state conditions, the microbial CUE is determined by the equation: soil CUE = 1 - (Rh / Dgr). In this equation, 'Rh' represents heterotrophic soil respiration. 'Dgr' stands for gross decomposition, which encompasses all carbon fluxes resulting from microbial decomposition activities. Under this definition of CUE, a microbial pool in the model is not required, making it applicable to both conventional soil carbon models and microbial explicit models. Here is the detailed calculation formula.

CUE definition in Manzoni et al. (2018) [1]:

CUE = BP / Cup; equation (1)

BP is biomass production and Cup is C uptake.

Biomass production is given by the balance of C uptake and carbon respired as CO_2:

BP = Cup - Rh; equation (2)

Putting equation (2) in equation (1) we get:

CUE = (Cup - Rh) / Cup = 1 - Resp / Cup; equation (3)

Within a soil model (e.g., CENTURY or MIMICS shown in Figure S1 a and b, respectively), at steady state, the C uptake of each pool equals the decomposition of that pool, which is also the pool's C flux. The steady-state assumption implies that microbial communities and SOC stock are stable in time (i.e. in equilibrium with boundary conditions). This is an approximation of real systems where SOC is subject to changes due to anthropogenic and natural changes (e.g., Holocene climatic variations). We assume that the path of CO_2 release in the model is regulated by microbes. Therefore, the CUE of the model (i.e., diagnosed CUE in the main text) is:

Cup = Dgr = C flux; equation (4)

Cup equals the sum of all C fluxes and also equal to the gross decomposition (Dgr). Gross decomposition refers to the sum of all C fluxes transferred among the modeled soil C pools that are mediated by microbial processes excluding physically or chemically mediated

transfers (e.g., sorption, aggregation, or leaching). It includes all C which is removed from organic matter pools, irrespectively if it is lost as CO_2 or transferred to another pool.

Putting equation (4) in equation (3) we get:

CUE = 1 - Rh/Dgr or **CUE = 1 - Rh/C flux.** equation (5)

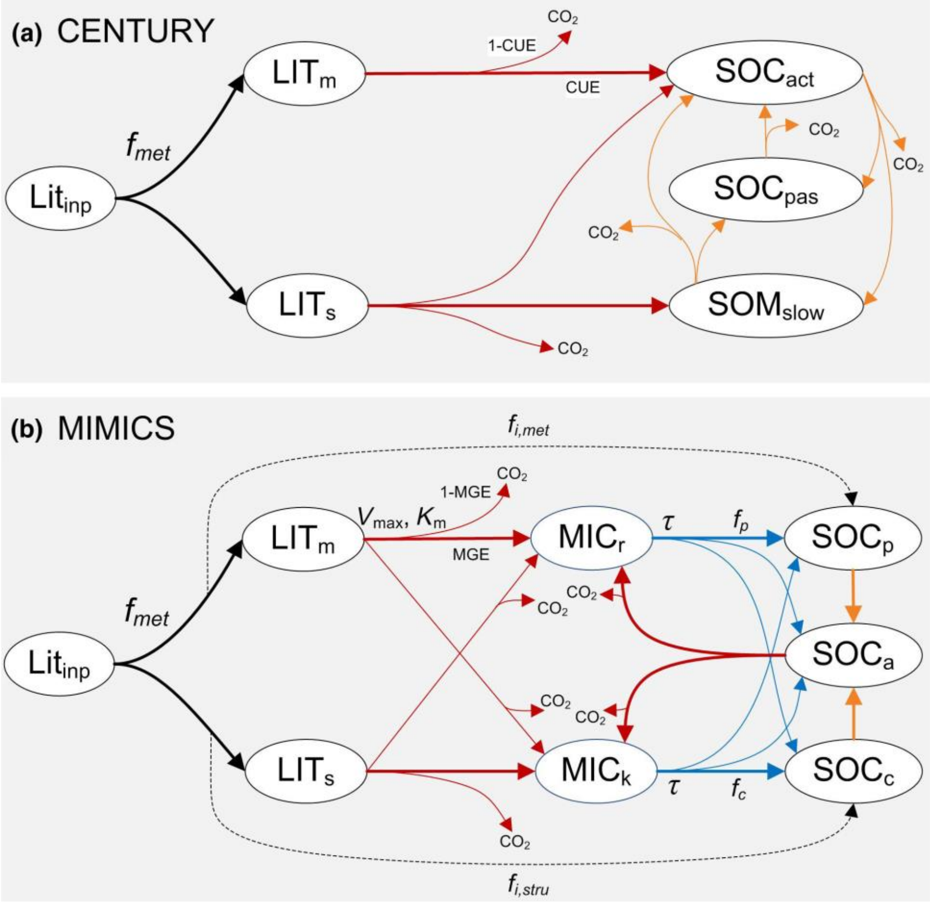

Figure S1. Framework of CENTURY model [2] **and MIcrobial-MIneral Carbon Stabilization (MIMICS) model** [3]**.** In the CENTURY model (a), decomposed C goes to soil carbon pools of increasing turnover time, respectively active C, slow C and passive C with a fraction of C lost as CO_2 upon transfer. In the MIMICS model (b), which explicitly considers microbial functional diversity by simulating two functional groups (MIC_r, inefficient, fast-growers; MIC_K, conservative, slow-growers) and their potential effects on litter decomposition and soil organic matter persistence (available SOM_a; chemically protected SOM_c; physically protected SOM_p). MGE:

Microbial Growth Efficiency parameter, analogous to CUE. τ: turnover rate of each pool. f: function calculating the fraction of the pool C that is transferred to another one. Paths illustrating microbial biomass turnover (blue paths) and those depicting the transfer of chemically recalcitrant or physically protected SOM into the available SOM pool (orange paths) were excluded from the calculation of diagnosed CUE, as these paths do not involve soil respiration losses. Figure taken from Zhang et al. (2020) [4].

Supplementary References:

1. Manzoni, S. *et al.* Reviews and syntheses: Carbon use efficiency from organisms to ecosystems – definitions, theories, and empirical evidence. *Biogeosciences* **15**, 5929–5949 (2018).
2. Parton, W. J., Stewart, J. W. B. & Cole, C. V. Dynamics of C, N, P and S in grassland soils: a model. *Biogeochemistry* **5**, 109–131 (1988).
3. Wieder, W. R., Grandy, A. S., Kallenbach, C. M., Taylor, P. G. & Bonan, G. B. Representing life in the Earth system with soil microbial functional traits in the MIMICS model. *Geosci. Model Dev.* **8**, 1789–1808 (2015).
4. Zhang, H. *et al.* Microbial dynamics and soil physicochemical properties explain large-scale variations in soil organic carbon. *Glob. Change Biol.* **26**, 2668–2685 (2020).
